# Health-related quality of life among Mongolian-speaking women with breast cancer receiving adjuvant endocrine therapy in China: a cross-sectional study

**DOI:** 10.1186/s41687-026-01101-5

**Published:** 2026-05-28

**Authors:** Rila Su, Claire Snyder, Laura Morlock, Albert W. Wu, Alden L. Gross, Jiafu Ji

**Affiliations:** 1 Department of Oncology, Inner Mongolia People’s Hospital, Hohhot, China; 2 Department of Health Policy and Management, Johns Hopkins University, Baltimore, USA; 3 Department of Health Policy and Management, Johns Hopkins University, Baltimore, USA; 4 Department of Epidemiology, Johns Hopkins University, Baltimore, USA; 5 State Key Laboratory of Holistic Integrative Management of Gastrointestinal Cancers, Peking University Cancer Hospital, Beijing, China

**Keywords:** Mongolian-speaking breast cancer patients, Health-related quality of life, Adjuvant endocrine therapy, FACT-ES

## Abstract

**Background:**

Breast cancer (BC) is the most common malignancy among women worldwide. Women in Mongolia, China, with BC face distinct socioeconomic and cultural barriers that may affect their health-related quality of life (HRQoL), particularly during adjuvant endocrine therapy (AET). This study assessed the HRQoL of Mongolian-speaking BC patients undergoing AET.

**Methodology:**

This descriptive, cross-sectional study included Mongolian-speaking BC patients from two hospitals in Inner Mongolia, China. Participants provided their sociodemographic and clinical information. They completed the Mongolian versions of the Functional Assessment of Cancer Therapy-Endocrine Symptoms (FACT-ES). Eastern Cooperative Oncology Group (ECOG) performance status was assessed by an oncologist during the clinical encounter and recorded for study purposes.

**Results:**

Of 215 patients approached, 200 participated and completed the questionnaires. Mean(SD) age = 55.45 ± 9.63 years; 72.5% were Stages I-II, 76% had a mastectomy, and 12% had breast-conserving surgery. Fourteen symptoms were reported by over 20% of participants. The most frequently reported were joint pain (82%), hot flashes (72%), cold sweats (61%) and mood swings (60%). Emotional well-being scores were lower than those reported in healthy females and female cancer patients, while functional well-being scores were relatively higher. Univariate analysis revealed significant differences in various subscales and overall FACT-ES scores across different characteristics, including annual income, occupational status, previous surgical procedures, cancer stage at diagnosis, and ECOG performance status (all *P*<.05). In multivariate regression analysis, ECOG performance status was negatively associated with most subscales and the overall FACT-ES (*P*<.001).

**Conclusions:**

These findings highlight the complex relationships between AET and HRQoL, as well as the specific challenges for Mongolian-speaking BC patients receiving AET in Inner Mongolia, China, underscoring the need for multidimensional approaches to improving HRQoL.

**Supplementary Information:**

The online version contains supplementary material available at 10.1186/s41687-026-01101-5.

## Background

Breast cancer (BC) is one of the most frequently diagnosed malignant tumors, with approximately 2.3 million new cases annually, accounting for 11.6% of all cancer cases [5, 27]. In China, BC is the most frequently diagnosed cancer in women, with approximately 367,900 new cases annually, comprising 8.6% of total cancer cases in 2018. While the mortality rate of BC decreased by 21% between 2000 and 2012 in the U.S., the BC mortality rate in China increased. This was likely due in part to lower early detection rates and substandard treatments [19].

According to data on BC incidence and mortality from the International Agency for Research on Cancer and the National Central Cancer Registry of China, there are several differences in BC patterns between China and the United States [15, 18]. One notable factor is the age of onset in BC. China has two age peaks: 45–55 years and 70–74 years. In comparison, there is a single peak in Europe and the U.S. between 60 and 64 years [15, 18]. Second, China established the one-child family planning policy in the 1970s, which decreased the fertility rate and increased the BC incidence rate [15, 18]. Finally, China has a large population of low-income and middle-income classes, but no nationwide BC screening program is currently in place [15, 18].

There are only a few studies on BC patterns and characteristics in Mongolia. One study compared Mongolian BC patients’ characteristics with Han BC patients in Inner Mongolia, Han BC patients from central China, and Chinese American BC patients [8]. There were no significant differences in age at onset, staging, and molecular subtypes of BC patients between the Mongolian and Han populations. However, differences were observed in comparison to Chinese American BC patients: over 60% of Chinese American BC patients were diagnosed between the ages of 55 and 69 at stage I, while 50% of Mongolian BC patients were diagnosed at ages 40–54 at Stage II [8]. In another review of BC patients in Mongolia, it was observed that the detected incidence of BC in Mongolia is the lowest in the world; however, mortality rates were increasing steadily due to a lack of BC screening associated with the country’s poor economic situation [14]. Although the risk factors of BC in Mongolia have been understudied, it is thought that Mongolians share the same risk factors as other Asian counterparts [14].

Adjuvant endocrine therapy (AET) is generally recommended for at least 5 years for hormone receptor positive BC patients to reduce the risk of recurrence and mortality [6, 20, 30, 33]. However, treatment adherence remains challenging, primarily due to treatment-related side effects, which decrease patient health-related quality of life (HRQoL [1, 20]. A U.S. study in a real-world clinical setting suggested that poor HRQoL during AET may increase the risk of underusing AET; the nonadherence rate was nearly 19% (211/1,114 [34]. Similarly, a retrospective study conducted among early-stage BC patients in Shanghai noted that the cumulative discontinuation rates among 706 patients gradually increased from the first to fifth year [44]. Another study in northeast China revealed that 258 (36.9%) of the BC patients did not complete AET [21]. Both studies suggest that AET nonadherence in China is not lower than in other countries. At the same time, the results indicated that in addition to treatment-related side effects, insurance coverage or financial restrictions also influenced nonadherence in BC patients [21, 44].

A Patient Reported Outcome (PRO) is a report of the patient’s health status that has not been interpreted or modified by the clinician or others [28]. Many questionnaires have been developed to assess patients’ HRQoL by capturing their functional status, symptoms, and well-being [12]. Patient-reported outcome measures (PROMs) are used to assess patients’ PROs, providing a valuable way to evaluate the impact and effectiveness of treatments from the patient’s perspective. Self-reported subjective experiences are the most reliable data source for HRQoL [24, 36]. Despite numerous HRQoL studies of people with BC, none has explicitly focused on Mongolian patients. BC patients in Inner Mongolia face unique healthcare challenges, including language barriers and limited access to culturally tailored health education, and have variable adherence to follow-up care. Assessing HRQoL in this population can help identify gaps in service delivery and inform culturally appropriate interventions. This study aimed to assess the HRQoL of Mongolian BC patients receiving AET in Inner Mongolia, China, and examine the associations between sociodemographic and clinical characteristics and HRQoL. We hypothesized that poorer clinical status and disadvantaged sociodemographic factors would be associated with poorer HRQoL scores.

## Methods

### Research design

This was a descriptive, cross-sectional study in a convenience sample of early-stage BC patients aged 18 years and older. Patients were recruited during September-October 2024 at the Oncology Department of Inner Mongolia People’s Hospital and Peking University Cancer Hospital (Inner Mongolia Campus)/Affiliated Cancer Hospital of Inner Mongolia Medical University. Data collection was conducted after obtaining ethical clearance from the Research Ethics Committee of Inner Mongolia People’s Hospital (202300610 K) and the Institutional Review Board of the Johns Hopkins Bloomberg School of Public Health (FWA #00000287). Verbal informed consent was obtained from all participants prior to data collection. This approach was approved by the institutional ethics committee of Inner Mongolia People’s Hospital due to the minimal-risk nature of the study and the use of anonymous, self-administered questionnaires. No identifiable personal information was collected, and participation was entirely voluntary. All participants could withdraw from the study at any time.

### Patient selection and data collection

Participants in this study were able to read and understand the Mongolian language (Mongolian script), and are referred to throughout as “Mongolian-speaking BC patients”. Patient selection was performed by screening Electronic Medical Records (EMRs) in the two hospitals’ Hospital Information System (HIS). Eligible Mongolian-speaking BC patients had completed their primary treatment with curative intent–which included some combination of surgery, adjuvant chemotherapy, and radiotherapy–and were recruited during follow-up visits while receiving AET. We excluded BC patients if they had a history of mental illness or cognitive impairment, were unwilling to participate, or had severe medical conditions, coexisting malignancies, or BC recurrence that could affect their self-reported HRQoL.

Participants were asked to respond to Mongolian versions of the Functional Assessment of Cancer Therapy-General (FACT-G) plus the Functional Assessment of Cancer Therapy-Endocrine Subscale (FACT-ES).

Researchers facilitated the process of the self-administered questionnaire by explaining its purpose and how participant input would be used. They offered clarification and assistance as needed when the participant had difficulty reading due to vision problems while avoiding influencing responses. Each entire session lasted approximately 15 min.

### Measures and variables

#### FACT-G [10]

The Functional Assessment of Cancer Therapy-General (FACT-G) provides a multidimensional measure of HRQoL and can be used in patients with any cancer. It consists of 27 items that measure four domains, including Physical Well-Being, Social/Family Well-Being, Emotional Well-Being, and Functional Well-Being. The FACT-G uses a 5-point Likert-type scale. The total score represents the sum of all items, with higher scores indicating better HRQoL. The FACT-G is translated and validated in more than 70 languages, including Mongolian, with satisfactory validity and reliability [22]. According to the FACIT measurement system guidance, a difference of 4–7% (3–7 units) in the FACT-G total score and a change of 7–11% (2–3 units) in the subscales represent clinically important differences [42].

#### FACT-ES

The FACT-ES questionnaire is designed for BC patients on endocrine therapy. It comprises 19 endocrine symptom subscale (ES) items, which can be combined with the FACT-G. When combined, the overall FACT-ES includes 46 questions, and the rules for scoring are calculated by summing the FACT-G and ES subscale scores, in accordance with FACIT scoring guidelines. This combined score provides an overall measure of HRQoL specific to patients receiving endocrine therapy. For subscales with missing items in FACT-G and FACT-ES, scores were prorated (Prorated Score = [Number of answered items /Sum of answered items]×Total number of items) if more than 50% of items in the subscale were completed; otherwise, the subscale score was treated as missing. Cases with excessive missing data were excluded from the relevant analyses [42]. The FACT-ES has been translated into more than 40 languages (including Mongolian) and overall reliability and validity were uniformly high(FACIT [17]; [32, 38]). Internal consistency reliability of the Mongolian version of the FACT-ES was assessed using Cronbach’s α and McDonald’s ω coefficients. The overall Cronbach’s α and McDonald’s ω coefficients were 0.92 and 0.93, respectively. For the subscales, Cronbach’s α coefficients ranged from 0.75 to 0.92, and McDonald’s ω coefficients ranged from 0.81 to 0.93, all exceeding the recommended threshold of 0.70, demonstrating good internal consistency across domains.

#### Eastern Cooperative Oncology Group (ECOG) performance status [39]

The ECOG Performance Status Scale describes a patient’s level of functioning in terms of their ability to care for themselves, daily activity, and physical ability (walking, working, etc.). Respondents rate items on a 5-point Likert scale: 0 = Fully active, able to carry on all pre-disease performance without restriction; 1 = Restricted in physically strenuous activity but ambulatory and able to carry out work of a light or sedentary nature, e.g., light housework, office work; 2 = Ambulatory and capable of all self-care but unable to carry out any work activities, up and about more than 50% of waking hours; 3 = Capable of only limited self-care, confined to bed or chair more than 50% of waking hours; 4 = Completely disabled, cannot carry on any self-care and totally confined to bed or chair; 5 = dead. ECOG performance status was assessed by an oncologist during the clinical encounter and recorded for study purposes.

### Data analysis

Participant sociodemographic data, clinical characteristics, symptoms, and HRQoL scores were summarized using descriptive statistics. For participant-level variables, missing data were minimal; where applicable, “unknown” categories were retained as valid categories rather than treated as missing. Continuous variables were reported as means±standard deviations and medians with ranges. Categorical variables were summarized as frequencies and percentages. Correlations between symptom levels and overall subscale scores were assessed using Pearson correlation coefficients. Univariable analyses were conducted to describe the associations between sociodemographic and clinical variables with HRQoL scores. Multivariable models were specified a priori based on clinical relevance and existing literature. To assess the robustness of the findings, alternative model specifications were explored by varying the inclusion of selected variables. These exploratory analyses did not materially change the direction or magnitude of the main associations. Data were analyzed using SPSS version 24 (SPSS Inc., Chicago, USA).

## Results

### Study participants

Of the patients screened, 215 met the study inclusion criteria, and 200 (93%) participated. The participants were mean age = 55.45, married (93.5%), urban residents (74.0%) with early-stage BC (72.5% Stage I-II), had undergone mastectomy (76.0%) and chemotherapy (93.0%), and were receiving aromatase inhibitor (AI) therapy (61.3%). The duration of AET at the time of assessment ranged from < 1 year to > 5 years (Table 1).

**Table 1:** Sociodemographic and clinical characteristics

Characteristics		*n*	%
Age (Years)	Mean ± SD = 55.45 ± 9.63, Median age: 56 (28–77)		
	< 55	88	44.0
	>=55	112	56.0
BMI	Mean ± SD = 24.85 ± 3.23, Median BMI: 24.65 (16.38–34.48)		
	< 18.5	3	1.5
	18.5–24	76	38.0
	> 24	121	60.5
Marital status	Married	187	93.5
	Single/widowed/separated/divorced	13	6.5
Education level	Primary school	51	25.5
	Middle school	106	53.0
	University	40	20.0
	Post-graduate	3	1.5
Occupation	Government/Official/Enterprise/ Business	99	49.5
	Self-employed/Farmer/Laborer	48	24.0
	Unemployed	53	26.5
Annual income	< 20,000 RMB	33	16.5
	20,000–50,000 RMB	75	37.5
	50,001-100,000 RMB	63	31.5
	100,001-200,000 RMB	26	13.0
	> 200,000 RMB	3	1.5
Residence	Urban	148	74.0
	Rural	52	26.0
Surgery	Mastectomy	152	76.0
	Breast-Conserving Therapy	24	12.0
	Mastectomy Reconstruction	2	1.0
	Unknown	20	10.0
	No Surgery	2	1.0
Chemotherapy	Yes	186	93.0
	No	14	7.0
Radiotherapy	Yes	77	38.5
	No	123	61.5
Stage at diagnosis	I	56	28.0
	II	89	44.5
	III	37	18.5
	Unknown	18	9.0
AET	Tamoxifen	51	25.5
	AIs	144	72.0
	Ovarian suppression	41	20.5
Time on AET	< 1 year	48	24.0
	1–2 years	50	25.0
	2.1-3 years	45	22.5
	3.1-4 years	29	14.5
	4.1-5 years	27	13.5
	> 5 years	1	0.5
Comorbidity	Yes	58	29.0
	No	142	71.0
ECOG Score	0	138	69.0
	1	52	26.0
	2	7	3.5
	3	3	1.5

BMI= body mass index, AET= adjuvant endocrine therapy, AIs= Aromatase inhibitors, ECOG=Eastern Cooperative Oncology Group; Note: ‘Unknown’ refers to participants who were unable to recall or specify the type of surgery they received and unable to specify their cancer stage at diagnosis, ‘No surgery’ refers to patients who did not undergo surgical treatment due to clinical or other reasons, AET categories were analyzed as non-mutually exclusive exposures because patients may receive combination or sequential endocrine therapies, so percentages reflect treatment prevalence rather than exclusive groups

### Description of endocrine treatment-related symptoms and HRQoL

**Fig. 1:**
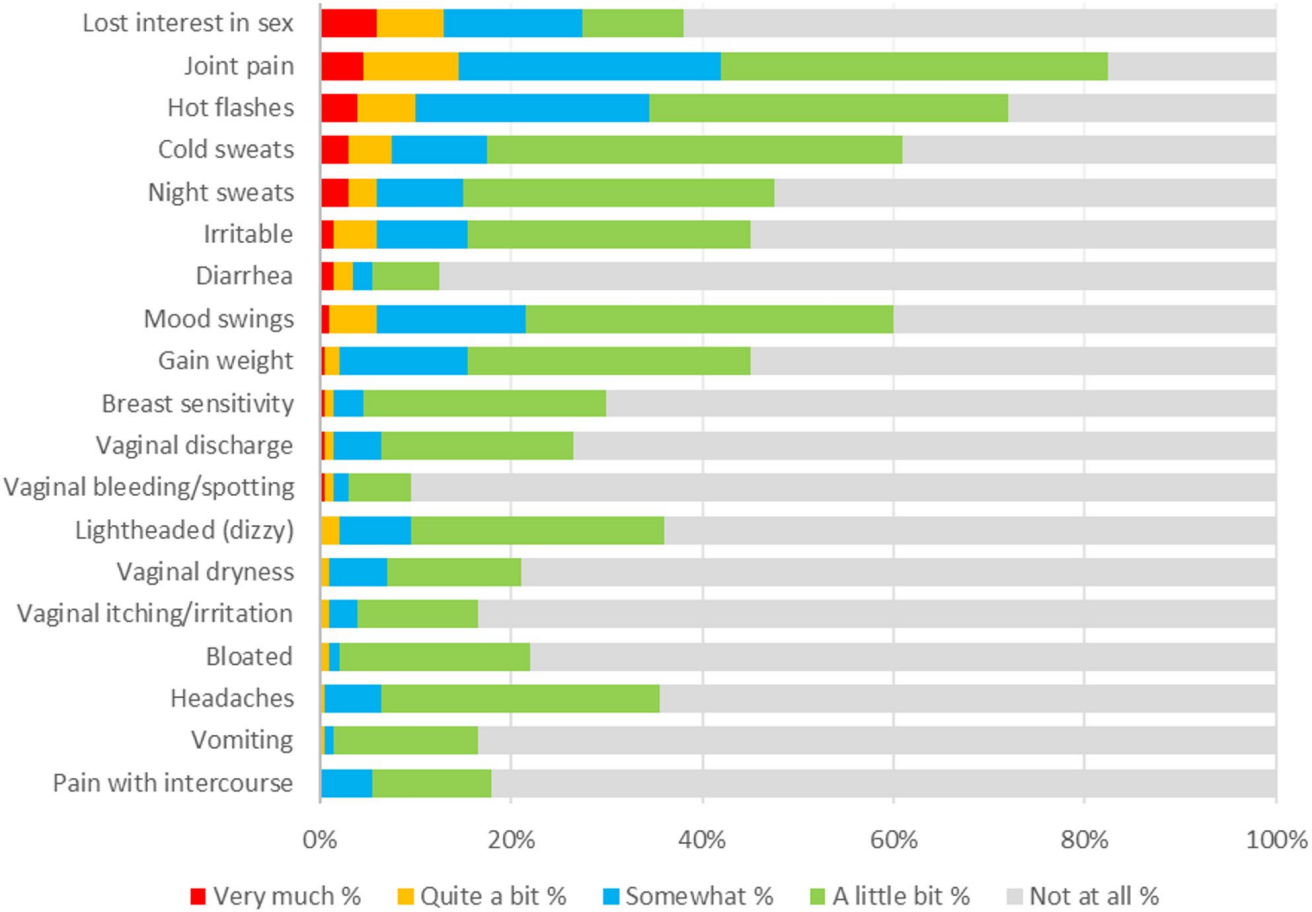
Summary of severity of AET symptoms (*N* = 200)

Nineteen symptoms from the FACT-ES were examined, and 14 were reported by more than 20% of participants (Fig. 1). These symptoms include joint pain (*n* = 165, 82.5%), hot flashes (*n* = 144, 72%), cold sweats (*n* = 122, 61%), mood swings (*n* = 120, 60%), night sweats (*n* = 95, 47.5%), weight gain (*n* = 90, 45%), irritability (*n* = 90, 45%), loss of interest in sex (*n* = 76, 38%), lightheadedness (*n* = 72, 36%), headaches (*n* = 71, 35.5%), breast sensitivity (*n* = 60, 30%), vaginal discharge (*n* = 53, 26.5%), bloating (*n* = 44, 22%), and vaginal dryness (*n* = 42, 21%). Only 3 symptoms were reported to be experienced “very much” or “quite a bit” by at least 10% of participants: joint pain (*n* = 29, 14.5%), loss of interest in sex (*n* = 26, 13%), and hot flashes (*n* = 20, 10%).

Table 2 compares the study population’s average scores on the FACT-G subscales and total score to healthy females and to female cancer patients in the U.S. and China. Study participants’ Emotional Well-Being was > 2 units worse than healthy women and female patients with other cancers in the U.S., which is at the threshold for clinical importance. Many experienced emotional strain, particularly ‘worry about disease progression‘(n = 111, 55.5%) and feelings of ‘nervousness‘(n = 90, 45%). The Functional Well-Being of study participants was the highest among all comparisons, with the difference exceeding the 2-unit clinical significance threshold except for the comparison with U.S. female patients with other cancers. Among the Functional Well-Being items, problems were most commonly noted for ‘I am sleeping well’ (n = 93, 46.5%) and ‘I am content with the quality of my life right now’ (n = 67, 33.5%) (Table S1).

**Table 2:** Comparisons of FACT-G scores among study participants, healthy women in the U.S. and China, and female cancer patients from the U.S. and China

Variable	Best Possible Score	Average in Current Study (*N* = 200)	FACT-G Study			
			Average Healthy Female (*N* = 544) U.S.^*^	Average Female Cancer Patients (*N* = 1,271) U.S.^*^	Average Healthy Female (*N* = 156) China^**^	Average Female Cancer Patients (*N* = 242) China^**^
PWB	28	22.54	22.10	21.60	23.23	20.03
SWB	28	21.66	19.80	22.30	23.19	22.11
EWB	24	16.64	19.40	18.70	17.48	17.67
FWB	28	20.45	18.30	19.50	17.02	16.94
Total	108	81.02	79.60	82.10	80.40	76.18

FACT-G=Functional Assessment of Cancer Therapy-General, EWB=emotional well-being, FWB=functional well-being, PWB=physical well-being, SWB= social/family well-being. ^*^ Based on the information from Brucker et al., [﻿^7^]. ^**^ Based on the information from Ding et al., [16]

### Relationships between symptoms and HRQoL

Of the 19 endocrine symptoms, 15 are statistically significantly correlated with the overall FACT-ES, 13 with Physical Well-Being, 6 with Social Well-Being, 8 with Emotional Well-Being, and 11 with Functional Well-Being (Table 3). Joint pain is moderately correlated with Physical Well-Being (*r*=.435), lost interest in sex with Social Well-Being (*r*=.470), and headaches with Functional Well-Being (*r*=.424). The symptoms most strongly correlated with Overall FACT-ES are lightheaded/dizzy (*r*=.452), joint pain (*r*=.448), headaches (*r*=.438), and lost interest in sex (*r*=.423), all indicating moderate correlations.

**Table 3:** Pearson correlations between symptoms, subscales, and overall FACT-ES

Symptom	PWB	SWB	EWB	FWB	Overall FACT-ES
Hot flashes	0.193^**^	0.022	0.140^*^	0.066	0.280^**^
Cold sweats	0.148^*^	-0.026	0.034	0.047	0.250^**^
Night sweats	0.087	-0.110	-0.012	-0.014	0.180^*^
Vaginal discharge	0.125	0.017	0.033	0.106	0.170^*^
Vaginal itching/irritation	0.159^*^	-0.018	-0.064	0.006	0.125
Vaginal bleeding/spotting	-0.016	-0.019	-0.029	-0.073	0.066
Vaginal dryness	0.057	0.092	0.016	0.020	0.115
Pain with intercourse	-0.036	0.016	0.006	-0.057	0.100
Lost interest in sex	0.258^**^	0.470^**^	0.220^**^	0.351^**^	0.423^**^
Gain weight	0.187^**^	0.142^*^	0.001	0.159^*^	0.243^**^
Lightheaded (dizzy)	0.361^**^	0.260^**^	0.263^**^	0.370^**^	0.452^**^
Vomiting	0.360^**^	0.139^*^	0.144^*^	0.289^**^	0.347^**^
Diarrhea	0.180^*^	0.132	0.049	0.170^*^	0.281^**^
Headaches	0.316^**^	0.141^*^	0.318^**^	0.424^**^	0.438^**^
Bloated	0.133	0.066	0.054	0.165^*^	0.215^**^
Breast sensitivity	0.305^**^	0.030	0.182^**^	0.186^**^	0.293^**^
Mood swings	0.185^**^	0.092	0.187^**^	0.299^**^	0.384^**^
Irritable	0.145^*^	0.134	0.114	0.229^**^	0.355^**^
Joint pain	0.435^**^	0.238^**^	0.189^**^	0.340^**^	0.448^**^

^*^P < .05, ^**^P < .01 EWB=emotional wellbeing, FWB=functional wellbeing, PWB=physical wellbeing, SWB= social/family wellbeing

### Univariable and multivariable analysis of Mongolian-speaking BC patients’ HRQoL

In univariable regression analysis (Table 4 and S2) unemployed participants had lower HRQoL scores across most subscales and overall FACT-ES compared with those employed in government or business sectors (*P*<.01). However, these associations were attenuated and largely not statistically significant in multivariable analyses. Additionally, HRQoL scores for both the subscales and the overall FACT-ES were higher among participants with higher annual income; those with an annual income exceeding 200,000 RMB show significant differences in most subscales and the overall FACT-ES when compared to the low-income group (< 20,000 RMB). Compared to the mastectomy group, the unknown surgery group showed higher scores in Functional Well-Being, Endocrine Symptoms, and overall FACT-ES in univariable analyses; however, this category likely reflects misclassification or recall bias and should be interpreted with caution. Additionally, as the cancer stage at diagnosis and ECOG performance status scores increase, HRQoL scores in most subscales and overall FACT-ES decrease significantly (*P*<.01). However, the association between duration of AET, AET treatment types, and HRQoL outcomes did not show statistically significant differences across subscales or total FACT-ES scores.

In multivariable analyses, stage III and poorer ECOG performance status consistently had the strongest negative associations with most subscales and overall FACT-ES(*P*<.01). Stage III was independently associated with poorer FACT-G and FACT-ES(*P*<.01). The ECOG performance score was strongly associated with most subscales, but scores of 1 and 2 were insignificant for Emotional Well-Being, and scores of 2 and 3 were insignificant for Endocrine Symptoms(*P*<.01) (Table 4 and S3). Age showed a small but significant association with the ES subscale, indicating a modest increase in endocrine symptoms as age increased (*P*<.05). Although AET duration and treatment type were included based on clinical relevance, they were not significantly associated with HRQoL outcomes in multivariable analyses.

**Table 4:** Univariable and Multivariable linear regression analysis of relationships between selected independent variables, FACT-G, FACT-ES and ES subscale

Dependent Independent	Univariable Model						Multivariable Model					
	FACT-G Unadjusted B (95% CI)	*P* value	FACT-ES Unadjusted B (95% CI)	*P* value	ES Unadjusted B (95% CI)	*P* value	FACT-G Adjusted B (95% CI)	*P* value	FACT-ES Adjusted B (95% CI)	*P* value	ES Adjusted B (95% CI)	*P* value
**Age**	-1.90(-0.42 ~ 0.04)	0.103	-0.18(-0.46 ~ 0.10)	0.208	0.01(-0.08 ~ 0.11)	0.808	-0.02(-0.25 ~ 0.20)	0.810	0.04(-0.19 ~ 0.35)	0.573	0.16(0.00 ~ 0.21)	**0.045**
**Occupational status** Government/Official/ Enterprise/Business (reference)												
Self-employed/Farmer/Laborer	-0.39(-5.75 ~ 4.97)	0.886	1.46(-5.11 ~ 8.02)	0.662	1.85(-0.37 ~ 4.07)	0.103	-0.10(-9.40 ~ 2.01)	0.203	-0.01(-7.37 ~ 6.53)	0.905	0.22(0.65 ~ 5.90)	**0.015**
Unemployed	-9.46(-14.64 ~ -4.27)	**0.000**	-9.26(-15.61 ~ -2.91)	**0.004**	0.19(-1.96 ~ 2.34)	0.859	-0.26(-15.46 ~ -3.09)	**0.003**	-0.16(-14.30 ~ 0.77)	0.078	0.17(-0.33 ~ 5.36)	0.083
**Annual income** < 20,000 (reference)												
20,000–50,000	2.46(-3.95 ~ 8.86)	0.450	2.51(-5.28 ~ 10.30)	0.526	0.05(-2.59 ~ 2.69)	0.970	-0.07(-7.84 ~ 3.48)	0.447	-0.03(-8.01 ~ 5.77)	0.749	0.08(-1.54 ~ 3.67)	0.420
50,001-100,000	8.32(1.73 ~ 14.91)	**0.014**	8.86(0.84 ~ 16.87)	**0.030**	0.54(-2.18 ~ 3.25)	0.698	-0.10(-10.68 ~ 4.16)	0.386	-0.01(-9.32 ~ 8.76)	0.951	0.22(-0.43 ~ 6.40)	0.086
100,001-200,000	0.15(-7.90 ~ 8.18)	0.972	0.25(-9.53 ~ 10.03)	0.96	0.10(-3.21 ~ 3.41)	0.951	-0.20(-18.34 ~ -0.77)	**0.033**	-0.11(-16.81 ~ 4.59)	0.261	0.18(-0.60 ~ 7.49)	0.094
> 200,000	22.27(3.79 ~ 40.76)	**0.018**	30.36(7.88 ~ 52.85)	**0.008**	8.09(0.48 ~ 15.70)	**0.037**	0.03(-12.24 ~ 20.62)	0.615	0.08(-7.39 ~ 32.63)	0.215	0.16(0.87 ~ 15.99)	**0.029**
**Cancer stage** I (reference)												
II	-6.21(-11.29 ~ -1.13)	**0.017**	-7.10(-13.27 ~ -0.93)	**0.024**	-0.89(-3.03 ~ 1.24)	0.410	-0.08(-7.07 ~ 1.95)	0.264	-0.06(-7.97 ~ 3.02)	0.375	0.94(-1.99 ~ 2.16)	0.936
III	-15.74(-22.04 ~ -9.43)	**0.000**	-18.74(-26.41 ~ -11.08)	**0.000**	-3.01(-5.66 ~ -0.35)	**0.027**	-0.27(-16.79 ~ -5.20)	**0.000**	-0.24(-18.79 ~ -4.67)	**0.001**	0.59(-340 ~ 1.93)	0.588
Unknown	-2.59(-10.65 ~ 5.48)	0.528	-1.39(-11.19 ~ 8.42)	0.781	1.20(-2.19 ~ 4.59)	0.486	0.06(-3.89 ~ 10.87)	0.352	0.10(-2.16 ~ 15.82)	0.136	0.05(-0.06 ~ 6.73)	0.054
**Surgical procedure** Mastectomy(reference)												
Breast Conservative Surgery	1.18(-5.66 ~ 8.03)	0.733	2.03(-6.23 ~ 10.30)	0.628	0.85(-1.91 ~ 3.60)	0.544	-0.00(-5.81 ~ 5.55)	0.963	0.00(-6.68 ~ 7.16)	0.946	0.02(-2.24 ~ 2.98)	0.780
Unknown	8.98(1.58 ~ 16.39)	**0.018**	12.58(3.64 ~ 21.53)	**0.006**	3.60(0.62 ~ 6.58)	**0.018**	0.11(-0.25 ~ 11.92)	0.060	0.14(1.71 ~ 16.54)	**0.016**	0.15(0.49 ~ 6.09)	**0.021**
Others	-3.32(-19.09 ~ 12.46)	0.679	-5.72(-24.77 ~ 13.34)	0.555	-2.40(-8.75 ~ 3.95)	0.457	-0.03(-17.50 ~ 10.63)	0.630	-0.04(-22.32 ~ 11.94)	0.551	-0.04(-8.23 ~ 4.71)	0.592
**ECOG performance status** ECOG 0 (reference)												
ECOG 1	-14.37(-18.75 ~ -9.99)	**0.000**	-19.80(-25.11 ~ -14.50)	**0.000**	-5.43(-7.36 ~ -3.51)	**0.000**	-0.32(-16.13 ~ -7.03)	**0.000**	-0.38(-22.43 ~ -11.34)	**0.000**	-0.36(-7.40 ~ -3.22)	**0.000**
ECOG 2	-23.43(-33.87 ~ -12.99)	**0.000**	-26.21(-38.84 ~ -13.57)	**0.000**	-2.78(-7.36 ~ 1.80)	0.233	-0.26(-33.41 ~ -12.27)	**0.000**	-0.25(-38.91 ~ -13.16)	**0.000**	-0.09(-8.06 ~ 1.67)	0.197
ECOG 3	-38.76(-54.48 ~ -23.04)	**0.000**	-38.16(-57.19 ~ -19.13)	**0.000**	0.60(-6.30 ~ 7.50)	0.864	-0.27(-50.08 ~ -19.27)	**0.000**	-0.22(-52.84 ~ -15.31)	**0.000**	0.01(-6.49 ~ 7.68)	0.868
**AET duration**	-0.81(-2.76 ~ 1.14)	0.413	-1.25(-3.62 ~ 1.12)	0.299	-0.44(-1.22 ~ 0.35)	0.272	-0.02(-1.91 ~ 1.50)	0.815	-0.02(-2.40 ~ 1.75)	0.757	-0.02(-0.91 ~ 0.66)	0.756

FACT-G=Functional Assessment of Cancer Therapy-General, FACT-ES =Functional Assessment of Cancer Therapy-Endocrine Subscale, ES=Endocrine Subscale, ECOG=Eastern Cooperative Oncology Group, Multivariable regression models were used to account for potential confounding by including relevant sociodemographic and clinical variables, Bold values indicate statistically significant P values(*P* < .05), R^2^ for final multivariable models FACT-G, FACT-ES and ES were 35.9%, 35.5% and 16.5% respectively

## Discussion

To our knowledge, this study provides the first comprehensive evaluation of HRQoL among Mongolian-speaking BC patients undergoing AET in China, revealing specific challenges that they experience. This study extends the PRO literature by providing evidence on HRQoL among Mongolian-speaking BC patients—a linguistically and culturally underrepresented population in PROM research. By applying a validated Mongolian version of the FACT-ES, this study contributes support to the cross-cultural applicability of PROMs and highlights the importance of language-appropriate tools in accurately capturing patient experiences. This study also identified key factors associated with domains of HRQoL and significant associations between HRQoL subscales and different characteristics. Understanding these factors may help inform supportive care strategies for HRQoL management.

The moderate associations between hot flashes and both Physical Well-Being and Functional Well-Being suggest that symptom control strategies may be considered important for maintaining daily functioning. The moderate associations between joint pain and dizziness with overall FACT-ES suggest that routine screening and individualized pain management may be helpful. The correlations between mood swings and irritability with both Functional Well-Being and overall FACT-ES point to the importance of integrating mental health support [3, 11, 32, 37, 41, 43]. Notably, sexual health’s association across all subscales indicates a potential unmet need for relational and sexual health counseling as part of survivorship care. In our study, the most serious and prevalent sexual symptom was loss of interest in sex. This issue was also observed in both a secondary data analysis conducted in China and a study carried out in Rome [25, 31], suggesting that the loss of sexual interest during AET may affect individuals in both lower and higher income countries. Addressing these symptoms through personalized management strategies, such as symptom monitoring, lifestyle interventions, and pharmacological approaches, may be associated with patient well-being [9, 32].

When compared with average healthy women and average female cancer patients in both the U.S. and China, Mongolian-speaking BC patients in the study reported lower Emotional Well-Being and higher Functional Well-Being. This pattern could be related to the psychological and social challenges that may reflect factors not directly measured in this study. However, similar studies have also shown that patients undertaking AET had lower Emotional Well-Being over the time points of follow-up; these studies utilized psychological assessment tools, like the Beck 13-item depression scale (BDI) and Symptom Checklist-90-Revised (SCL-90-R) along with FACT-ES or EORTC QLQ-C30 to identify the patients’ psychological changes during AET [4, 35]. As the FACT-G represents the core generic HRQoL instrument across cancer types, comparisons were made based on the FACT-G subscale and total scores, which are conceptually and structurally comparable across different cancer populations. However, the differences in patient populations, disease characteristics, treatment contexts, and cultural settings should be considered. Therefore, these comparisons are intended to provide descriptive context rather than direct analytical equivalence.

Occupational status was associated with HRQoL in univariable analyses, but these associations were attenuated after adjustment. The unemployed had poorer HRQoL, particularly in Physical Well-Being, Social Well-Being, and Emotional Well-Being. These findings highlight the vulnerability of economically disadvantaged patients, consistent with prior findings in cancer populations [23, 26]. Women engaged in stable occupations tend to benefit from better resources and social support, which can contribute to improved HRQoL. Employment and economic stability can serve as protective factors in cancer recovery [29].

ECOG performance emerged as the strongest factor, with poorer functional status correlating with lower scores across all domains, suggesting the need for rehabilitation programs aimed at improving functional capacity [9, 32]. Surgical procedures were not significantly associated with HRQoL in adjusted analyses, and the “unknown” category should be interpreted with caution due to potential misclassification or recall bias; in other studies, their impact varied and depended on survey timing and patient demographics [2, 13, 40]. While surgical intervention is a critical component of BC management, its impact on HRQoL may be mediated by post-treatment care and psychological adaptation, and this may reflect factors not directly measured in this study. Different AET modalities have distinct side-effect profiles and are associated with different patient characteristics (e.g., menopausal status), but we did not observe significant differences in HRQoL across treatment types in this study. These results may be related to limited sample sizes within each duration subgroup, which reduced statistical power to detect differences. Patients who experienced severe side effects and discontinued treatment would not be reflected in our sample. As this was a cross-sectional study, HRQoL was assessed at a single time point for each participant, which limits the ability to capture longitudinal changes from diagnosis through different phases of AET. Future longitudinal studies are needed to better characterize trajectories of HRQoL from diagnosis through initiation and continuation of AET, and to identify critical periods where interventions may be most beneficial. In addition, larger sample sizes are needed to further explore potential differences in HRQoL across specific endocrine therapy regimens and stratify analyses by menopausal status and treatment duration. Overall, these findings underscore the complex relationships between AET and HRQoL and the specific challenges faced by Mongolian-speaking women with BC receiving AET in China.

Understanding the HRQoL of Mongolian-speaking BC patients receiving AET allows clinicians to better tailor symptom management, patient education, and supportive care strategies for this linguistic and cultural subgroup. This may be associated with improved outcomes in this study population. The strong association between ECOG performance status and HRQoL underscores the importance of routine functional assessment in clinical care. Integrating HRQoL monitoring with performance status evaluation may help clinicians identify patients at risk of poor outcomes and guide targeted interventions. The high prevalence of endocrine-related symptoms, particularly joint pain and vasomotor symptoms, suggests a need for proactive symptom management strategies, including patient education, pharmacologic management, and supportive care interventions to improve treatment adherence. However, limitations include a cross-sectional design that limits causal inferences, and a small sample size that may reduce the generalizability of our findings. The small number of participants with more extended AET usage may have limited our ability to observe associations between AET usage duration and HRQoL. Furthermore, the absence of a follow-up component in our study means that we were unable to track changes over time. Notably, this study included only patients who were actively receiving AET and attending follow-up visits, while excluding those with recurrence, distant metastasis, severe illness, or treatment discontinuation. As a result, patients with the poorest HRQoL—particularly those who discontinued AET due to severe side effects or disease progression may be underrepresented. This may have led to an overestimation of HRQoL in the study population. Future studies should include patients who have discontinued AET, as well as those with more advanced diseases, to better capture the full range of treatment experiences and to understand the relationship between treatment discontinuation and HRQoL. While comparisons with other populations provide valuable context, differing healthcare systems and cultural norms may complicate direct comparisons. Future studies should adopt longitudinal designs to better understand the dynamics of HRQoL and explore targeted interventions for this population.

## Conclusions

This cross-sectional study describes HRQoL among Mongolian-speaking breast cancer patients receiving AET in Inner Mongolia, China, and identifies sociodemographic and clinical factors associated with HRQoL. Poorer functional status, unemployment, and more advanced disease stage were associated with lower HRQoL. These findings highlight the importance of considering functional status, socioeconomic context, and culturally appropriate care when addressing HRQoL in this population. Further longitudinal studies are needed to better understand changes in HRQoL over time and to inform targeted interventions.

## Supplementary Information

Below is the link to the electronic supplementary material.

Supplementary Material 1

## Data Availability

The datasets used and/or analysed during the current study are available from the corresponding author on reasonable request.

## Abbreviations

HRQoL: Health-Related Quality of Life
BC: Breast Cancer
AET: Adjuvant Endocrine Therapy
FACT-G: Functional Assessment of Cancer Therapy-General
ES: Endocrine Subscale
FACT-ES: Functional Assessment of Cancer Therapy-Endocrine Subscale
ECOG: Eastern Cooperative Oncology Group
AIs: Aromatase Inhibitors
PRO: Patient Reported Outcome
PROMs: Patient-Reported Outcome Measures
PWB: Physical Well-Being
SWB: Social/Family Well-Being
EWB: Emotional Well-Being
FWB: Functional Well-Being
BMI: Body Mass Index
BDI: Beck's 13-item Depression scale
SCL-90-R: Symptom Checklist-90-Revised
