# Supplementary material for: Health-related quality of life among Mongolian-speaking women with breast cancer receiving adjuvant endocrine therapy in China: a cross-sectional study
Source: J Patient Rep Outcomes. 2026 May 28;10:129. doi: 10.1186/s41687-026-01101-5 (PMC13424016; doi:10.1186/s41687-026-01101-5)
Supplement: Supplementary file 1 — Supplementary Material 1 [file 41687_2026_1101_MOESM1_ESM.docx]

Supplementary Tables

S1. Summary of FACT-G item responses

| No. | Items | Not at all | | A little bit | | Somewhat | | Quite a bit | | Very much | | Scoring |
| --- | --- | --- | --- | --- | --- | --- | --- | --- | --- | --- | --- | --- |
|  |  | n | % | n | % | n | % | n | % | n | % | Reverse scoring |
| GP1 | Lack of energy | 132 | 66.0% | 26 | 13.0% | 33 | 16.5% | 6 | 3.0% | 3 | 1.5% |  |
| GP2 | Nausea | 152 | 76.0% | 37 | 18.5% | 6 | 3.0% | 3 | 1.5% | 2 | 1.0% |  |
| GP3 | Trouble to meet family needs | 153 | 76.5% | 31 | 15.5% | 7 | 3.5% | 7 | 3.5% | 2 | 1.0% |  |
| GP4 | Pain | 73 | 36.5% | 79 | 39.5% | 33 | 16.5% | 15 | 7.5% | - | - |  |
| GP5 | Side effect | 49 | 24.5% | 88 | 44.0% | 46 | 23.0% | 13 | 6.5% | 4 | 2.0% |  |
| GP6 | Feel ill | 42 | 21.0% | 54 | 27.0% | 60 | 30.0% | 31 | 15.5% | 13 | 6.5% |  |
| GP7 | Spend time in bed | 179 | 89.5% | 13 | 6.5% | 4 | 2.0% | 3 | 1.5% | - | - |  |
| GS1 | Close to friends | - | - | 1 | 0.5% | 3 | 1.5% | 54 | 27.0% | 142 | 71.0% |  |
| GS2 | Emotional support from family | - | - | - | - | 5 | 2.5% | 52 | 26.0% | 143 | 71.5% |  |
| GS3 | Emotional support from friends | - | - | - | - | 5 | 2.5% | 56 | 28.0% | 139 | 69.5% |  |
| GS4 | Family acceptance | - | - | 11 | 5.5% | 29 | 14.5% | 80 | 40.0% | 80 | 40.0% |  |
| GS5 | Family communication | 68 | 34.0% | 42 | 21.0% | 29 | 14.5% | 29 | 14.5% | 32 | 16.0% |  |
| GS6 | Close to partner | 2 | 1.0% | 2 | 1.0% | 2 | 1.0% | 75 | 37.5% | 119 | 59.5% |  |
| GS7 | Satisfaction with sex life | 43 | 21.5% | 15 | 7.5% | 33 | 16.5% | 45 | 22.5% | 64 | 32.0% |  |
| GE1 | Feel sad | 71 | 35.5% | 80 | 40.0% | 32 | 16.0% | 14 | 7.0% | 3 | 1.5% | Reverse scoring |
| GE2 | Satisfaction with coping illness | 20 | 10.0% | 19 | 9.5% | 42 | 21.0% | 72 | 36.0% | 47 | 23.5% |  |
| GE3 | Losing hope | 122 | 61.0% | 51 | 25.5% | 19 | 9.5% | 6 | 3.0% | 2 | 1.0% | Reverse scoring |
| GE4 | Nervous | 44 | 22.0% | 66 | 33.0% | 43 | 21.5% | 39 | 19.5% | 8 | 4.0% |  |
| GE5 | Worry about dying | 77 | 38.5% | 74 | 37.0% | 33 | 16.5% | 11 | 5.5% | 5 | 2.5% |  |
| GE6 | Worry condition gets worse | 31 | 15.5% | 58 | 29.0% | 41 | 20.5% | 48 | 24.0% | 22 | 11.0% |  |
| GF1 | Able to work | 1 | 0.5% | 8 | 4.0% | 22 | 11.0% | 71 | 35.5% | 98 | 49.0% |  |
| GF2 | Work is fulfilling | 1 | 0.5% | 8 | 4.0% | 32 | 16.0% | 71 | 35.5% | 88 | 44.0% |  |
| GF3 | Enjoy life | 2 | 1.0% | 8 | 4.0% | 42 | 21.0% | 75 | 37.5% | 73 | 36.5% |  |
| GF4 | Acceptance of illness | 1 | 0.5% | 15 | 7.5% | 63 | 31.5% | 75 | 37.5% | 46 | 23.0% |  |
| GF5 | Sleeping well | 14 | 7.0% | 18 | 9.0% | 61 | 30.5% | 76 | 38.0% | 31 | 15.5% |  |
| GF6 | Enjoying things | 1 | 0.5% | 8 | 4.0% | 42 | 21.0% | 100 | 50.0% | 49 | 24.5% |  |
| GF7 | Content with current QoL | 5 | 2.5% | 15 | 7.5% | 47 | 23.5% | 84 | 42.0% | 49 | 24.5% |  |

GP=general physical, GS=general social, GE=general emotional, GF=general functional

S2. Univariable linear regression analysis of potential influencing factors on each subscale and overall FACT-ES

| Potential influencing factors for Physical well-being | Unstandardized Coefficients | | Standardized Coefficients | t | P-value |
| --- | --- | --- | --- | --- | --- |
|  | β | Std.Error | β |  |  |
| Constants | 23.01 | 0.424 | - | 54.254 | 0.000 |
| Self-employed/Farmer/Laborer vs Government/official/enterprise/ business | 0.157 | 0.742 | 0.016 | 0.211 | 0.833 |
| Unemployed vs Government/official/enterprise/ business | -1.916 | 0.718 | -0.198 | -2.667 | 0.008** |
| Constants | 21.818 | 0.741 | - | 29.437 | 0.000 |
| 20,000-50,000 RMB vs <20,000 | 0.582 | 0.889 | 0.066 | 0.654 | 0.514 |
| 50,001-100,000 RMB vs <20,000 | 1.261 | 0.915 | 0.137 | 1.378 | 0.170 |
| 100,001-200,000 RMB vs <20,000 | 0.143 | 1.117 | 0.011 | 0.128 | 0.898 |
| >200,000 RMB vs <20,000 | 5.848 | 2.568 | 0.166 | 2.278 | 0.024* |
| Constants | 22.289 | 0.345 | - | 64.626 | 0.000 |
| Breast-conserving therapy vs mastectomy | 0.919 | 0.934 | 0.07 | 0.984 | 0.326 |
| Unknown vs. mastectomy | 1.961 | 1.011 | 0.137 | 1.938 | 0.054 |
| Others vs. mastectomy | -2.789 | 2.154 | -0.091 | -1.295 | 0.197 |
| Constants | 23.929 | 0.559 | - | 42.82 | 0.000 |
| II vs I | -1.547 | 0.713 | -0.18 | -2.168 | 0.031* |
| III vs I | -3.199 | 0.886 | -0.29 | -3.611 | 0.000** |
| Unknown vs. I | -1.206 | 1.133 | -0.081 | -1.065 | 0.288 |
| Constants | 23.841 | 0.316 | - | 75.514 | 0.000 |
| ECOG 1 vs ECOG 0 | -3.398 | 0.603 | -0.348 | -5.631 | 0.000** |
| ECOG 2 vs ECOG 0 | -7.841 | 1.437 | -0.337 | -5.457 | 0.000** |
| ECOG 3 vs ECOG 0 | -9.507 | 2.164 | -0.27 | -4.393 | 0.000** |
| Potential influencing factors for Social/Family well-being | Unstandardized Coefficients | | Standardized Coefficients | t | P-value |
|  | β | Std.Error | β |  |  |
| Constants | 22.253 | 0.386 | - | 57.604 | 0.000 |
| Self-employed/Farmer/Laborer vs Government/official/enterprise/ business | 0.164 | 0.676 | 0.018 | 0.243 | 0.808 |
| Unemployed vs Government/official/enterprise/ business | -2.385 | 0.654 | -0.265 | -3.645 | 0.000** |
| Constants | 20.212 | 0.682 | - | 29.637 | 0.000 |
| 20,000-50,000 RMB vs <20,000 | 1.188 | 0.818 | 0.145 | 1.451 | 0.148 |
| 50,001-100,000 RMB vs <20,000 | 2.375 | 0.842 | 0.278 | 2.821 | 0.005** |
| 100,001-200,000 RMB vs <20,000 | 1.48 | 1.027 | 0.126 | 1.441 | 0.151 |
| >200,000 RMB vs <20,000 | 4.121 | 2.362 | 0.126 | 1.744 | 0.083 |
| Constants | 21.559 | 0.321 | - | 67.092 | 0.000 |
| Breast-conserving therapy vs mastectomy | -0.476 | 0.87 | -0.039 | -0.547 | 0.585 |
| Unknown vs. mastectomy | 1.741 | 0.942 | 0.132 | 1.847 | 0.066 |
| Others vs. mastectomy | -0.809 | 2.007 | -0.029 | -0.403 | 0.687 |
| Constants | 23.018 | 0.515 | - | 44.663 | 0.000 |
| II vs I | -1.4 | 0.658 | -0.175 | -2.128 | 0.035* |
| III vs I | -3.18 | 0.817 | -0.311 | -3.892 | 0.000** |
| Unknown vs. I | -1.629 | 1.045 | -0.118 | -1.559 | 0.121 |
| Constants | 22.891 | 0.293 | - | 78.26 | 0.000 |
| ECOG 1 vs ECOG 0 | -3.314 | 0.559 | -0.367 | -5.928 | 0.000** |
| ECOG 2 vs ECOG 0 | -6.606 | 1.331 | -0.306 | -4.962 | 0.000** |
| ECOG 3 vs ECOG 0 | -9.225 | 2.005 | -0.283 | -4.6 | 0.000** |
| Potential influencing factors for Emotional well-being | Unstandardized Coefficients | | Standardized Coefficients | t | P-value |
|  | β | Std.Error | β |  |  |
| Constants | 17.343 | 0.513 | - | 33.797 | 0.000 |
| Self-employed/Farmer/Laborer vs Government/official/enterprise/ business | -0.281 | 0.898 | -0.023 | -0.313 | 0.755 |
| Unemployed vs Government/official/enterprise/ business | -2.4 | 0.869 | -0.205 | -2.762 | 0.006** |
| Constants | 16.212 | 0.875 | - | 18.527 | 0.000 |
| 20,000-50,000 RMB vs <20,000 | 0.188 | 1.05 | 0.018 | 0.179 | 0.858 |
| 50,001-100,000 RMB vs <20,000 | 1.883 | 1.08 | 0.169 | 1.743 | 0.083 |
| 100,001-200,000 RMB vs <20,000 | -2.404 | 1.318 | -0.156 | -1.824 | 0.07 |
| >200,000 RMB vs <20,000 | 5.121 | 3.031 | 0.12 | 1.689 | 0.093 |
| Constants | 16.447 | 0.419 | - | 39.207 | 0.000 |
| Breast-conserving therapy vs mastectomy | -0.447 | 1.136 | -0.028 | -0.394 | 0.694 |
| Unknown vs. mastectomy | 2.253 | 1.23 | 0.131 | 1.831 | 0.069 |
| Others vs. mastectomy | 1.053 | 2.62 | 0.029 | 0.402 | 0.688 |
| Constants | 18.071 | 0.667 | - | 27.109 | 0.000 |
| II vs I | -1.352 | 0.851 | -0.13 | -1.589 | 0.114 |
| III vs I | -4.396 | 1.057 | -0.33 | -4.159 | 0.000** |
| Unknown vs. I | -0.183 | 1.352 | -0.01 | -0.135 | 0.893 |
| Constants | 17.486 | 0.429 | - | 40.793 | 0.000 |
| ECOG 1 vs ECOG 0 | -2.409 | 0.819 | -0.204 | -2.94 | 0.004** |
| ECOG 2 vs ECOG 0 | -3.343 | 1.951 | -0.119 | -1.713 | 0.088 |
| ECOG 3 vs ECOG 0 | -6.819 | 2.939 | -0.16 | -2.32 | 0.021* |
| Potential influencing factors for Functional well-being | Unstandardized Coefficients | | Standardized Coefficients | t | P-value |
|  | β | Std.Error | β |  |  |
| Constants | 21.283 | 0.512 | - | 41.533 | 0.000 |
| Self-employed/Farmer/Laborer vs Government/official/enterprise/ business | -0.429 | 0.897 | -0.035 | -0.478 | 0.633 |
| Unemployed vs Government/official/enterprise/ business | -2.755 | 0.868 | -0.234 | -3.174 | 0.002** |
| Constants | 19.152 | 0.885 | - | 21.639 | 0.000 |
| 20,000-50,000 RMB vs <20,000 | 0.502 | 1.062 | 0.047 | 0.472 | 0.637 |
| 50,001-100,000 RMB vs <20,000 | 2.801 | 1.093 | 0.251 | 2.564 | 0.011* |
| 100,001-200,000 RMB vs <20,000 | 0.925 | 1.333 | 0.06 | 0.694 | 0.488 |
| >200,000 RMB vs <20,000 | 7.182 | 3.066 | 0.168 | 2.342 | 0.020* |
| Constants | 20.02 | 0.418 | - | 47.87 | 0.000 |
| Breast-conserving therapy vs mastectomy | 1.189 | 1.133 | 0.074 | 1.05 | 0.295 |
| Unknown vs. mastectomy | 3.03 | 1.226 | 0.175 | 2.471 | 0.014* |
| Others vs. mastectomy | -0.77 | 2.612 | -0.021 | -0.295 | 0.769 |
| Constants | 22.179 | 0.658 | - | 33.706 | 0.000 |
| II vs I | -1.909 | 0.84 | -0.183 | -2.273 | 0.024* |
| III vs I | -4.962 | 1.043 | -0.371 | -4.757 | 0.000** |
| Unknown vs. I | 0.433 | 1.334 | 0.024 | 0.324 | 0.746 |
| Constants | 22.21 | 0.377 | - | 58.988 | 0.000 |
| ECOG 1 vs ECOG 0 | -5.249 | 0.72 | -0.443 | -7.293 | 0.000** |
| ECOG 2 vs ECOG 0 | -5.639 | 1.714 | -0.2 | -3.29 | 0.001** |
| ECOG 3 vs ECOG 0 | -13.21 | 2.581 | -0.309 | -5.118 | 0.000** |
| Potential influencing factors for Endocrine symptoms | Unstandardized Coefficients | | Standardized Coefficients | t | P-value |
|  | β | Std.Error | β |  |  |
| Constants | 65.071 | 0.643 | - | 101.167 | 0.000 |
| Self-employed/Farmer/Laborer vs Government/official/enterprise/ business | 1.846 | 1.126 | 0.123 | 1.64 | 0.103 |
| Unemployed vs Government/official/enterprise/ business | 0.193 | 1.089 | 0.013 | 0.178 | 0.859 |
| Constants | 65.242 | 1.114 | - | 58.547 | 0.000 |
| 20,000-50,000 RMB vs <20,000 | 0.051 | 1.337 | 0.004 | 0.038 | 0.97 |
| 50,001-100,000 RMB vs <20,000 | 0.535 | 1.376 | 0.039 | 0.389 | 0.698 |
| 100,001-200,000 RMB vs <20,000 | 0.104 | 1.679 | 0.005 | 0.062 | 0.951 |
| >200,000 RMB vs <20,000 | 8.091 | 3.86 | 0.154 | 2.096 | 0.037* |
| Constants | 65.151 | 0.516 | - | 126.36 | 0.000 |
| Breast-conserving therapy vs mastectomy | 0.849 | 1.396 | 0.043 | 0.608 | 0.544 |
| Unknown vs. mastectomy | 3.599 | 1.512 | 0.169 | 2.38 | 0.018* |
| Others vs. mastectomy | -2.401 | 3.22 | -0.053 | -0.746 | 0.457 |
| Constants | 66.411 | 0.848 | - | 78.294 | 0.000 |
| II vs I | -0.894 | 1.083 | -0.069 | -0.826 | 0.41 |
| III vs I | -3.005 | 1.345 | -0.182 | -2.235 | 0.027* |
| Unknown vs. I | 1.2 | 1.72 | 0.054 | 0.698 | 0.486 |
| Constants | 67.065 | 0.51 | - | 131.394 | 0.000 |
| ECOG 1 vs ECOG 0 | -5.431 | 0.976 | -0.372 | -5.566 | 0.000** |
| ECOG 2 vs ECOG 0 | -2.78 | 2.323 | -0.08 | -1.196 | 0.233 |
| ECOG 3 vs ECOG 0 | 0.601 | 3.499 | 0.011 | 0.172 | 0.864 |
| Potential influencing factors for FACT-ES | Unstandardized Coefficients | | Standardized Coefficients | t | P-value |
|  | β | Std.Error | β |  |  |
| Constants | 148.96 | 1.902 | - | 78.309 | 0.000 |
| Self-employed/Farmer/Laborer vs Government/official/enterprise/ business | 1.457 | 3.329 | 0.032 | 0.438 | 0.662 |
| Unemployed vs Government/official/enterprise/ business | -9.261 | 3.221 | -0.212 | -2.875 | 0.004** |
| Constants | 142.636 | 3.291 | - | 43.337 | 0.000 |
| 20,000-50,000 RMB vs <20,000 | 2.51 | 3.95 | 0.063 | 0.636 | 0.526 |
| 50,001-100,000 RMB vs <20,000 | 8.856 | 4.063 | 0.213 | 2.18 | 0.030* |
| 100,001-200,000 RMB vs <20,000 | 0.248 | 4.958 | 0.004 | 0.05 | 0.96 |
| >200,000 RMB vs <20,000 | 30.364 | 11.402 | 0.191 | 2.663 | 0.008** |
| Constants | 145.467 | 1.547 | - | 94.018 | 0.000 |
| Breast-conserving therapy vs mastectomy | 2.033 | 4.19 | 0.034 | 0.485 | 0.628 |
| Unknown vs. mastectomy | 12.583 | 4.537 | 0.196 | 2.773 | 0.006** |
| Others vs. mastectomy | -5.717 | 9.662 | -0.042 | -0.592 | 0.555 |
| Constants | 153.607 | 2.451 | - | 62.675 | 0.000 |
| II vs I | -7.102 | 3.128 | -0.183 | -2.27 | 0.024* |
| III vs I | -18.742 | 3.886 | -0.378 | -4.824 | 0.000** |
| Unknown vs. I | -1.385 | 4.969 | -0.021 | -0.279 | 0.781 |
| Constants | 153.493 | 1.407 | - | 109.057 | 0.000 |
| ECOG 1 vs ECOG 0 | -19.8 | 2.69 | -0.451 | -7.36 | 0.000** |
| ECOG 2 vs ECOG 0 | -26.207 | 6.406 | -0.25 | -4.091 | 0.000** |
| ECOG 3 vs ECOG 0 | -38.159 | 9.649 | -0.241 | -3.955 | 0.000** |

*P<0.05, **P<0.01

S3. Multivariable linear regression analysis of relationships between selected independent variables, each subscale and overall FACT-ES

| **Potential influencing factors for Physical well-being** | **B** | **Beta** | **Std.Err** | ***t*** | **P** | **95% CI** |
| --- | --- | --- | --- | --- | --- | --- |
| Constants | 25.794 | - | 1.116 | 23.109 | 0.000 | 23.606 ~ 27.981 |
| Government/official/enterprise/ business | - | - | - | - | - | - |
| Self-employed/Farmer/Laborer | -0.832 | -0.083 | 0.796 | -1.046 | 0.297 | -2.391 ~ 0.727 |
| Unemployed | -1.945 | -0.201 | 0.833 | -2.334 | 0.021* | -3.578 ~ -0.311 |
| < 20,000 RMB | - | - | - | - | - | - |
| 20,000-50,000 RMB | -0.642 | -0.073 | 0.792 | -0.811 | 0.418 | -2.193 ~ 0.909 |
| 50,001-100,000 RMB | -1.472 | -0.160 | 1.011 | -1.456 | 0.147 | -3.455 ~ 0.510 |
| 100,001-200,000 RMB | -2.053 | -0.161 | 1.185 | -1.732 | 0.085 | -4.375 ~ 0.270 |
| >200,000 RMB | 1.659 | 0.047 | 2.351 | 0.706 | 0.481 | -2.949 ~ 6.268 |
| Mastectomy | - | - | - | - | - | - |
| Breast-conserving therapy | 0.803 | 0.061 | 0.814 | 0.987 | 0.325 | -0.792 ~ 2.399 |
| Unknown | 1.182 | 0.083 | 0.873 | 1.354 | 0.177 | -0.529 ~ 2.893 |
| Others | -1.685 | -0.055 | 1.920 | -0.877 | 0.381 | -5.449 ~ 2.079 |
| I | - | - | - | - | - | - |
| II | -0.612 | -0.071 | 0.638 | -0.960 | 0.339 | -1.862 ~ 0.638 |
| III | -2.163 | -0.196 | 0.828 | -2.614 | 0.010** | -3.786 ~ -0.541 |
| Unknown | 0.641 | 0.043 | 1.040 | 0.616 | 0.538 | -1.398 ~ 2.680 |
| ECOG 0 | - | - | - | - | - | - |
| ECOG 1 | -2.879 | -0.295 | 0.627 | -4.589 | 0.000** | -4.108 ~ -1.649 |
| ECOG 2 | -7.779 | -0.334 | 1.491 | -5.219 | 0.000** | -10.701 ~ -4.857 |
| ECOG 3 | -9.161 | -0.260 | 2.187 | -4.189 | 0.000** | -13.448 ~ -4.875 |
| **Potential influencing factors for Social well-being** | **B** | **Beta** | **Std.Err** | ***t*** | **P** | **95% CI** |
| Constants | 23.799 | - | 1.034 | 23.014 | 0.000 | 21.772 ~ 25.826 |
| Government/official/enterprise/ business | - | - | - | - | - | - |
| Self-employed/Farmer/Laborer | -0.196 | -0.021 | 0.737 | -0.266 | 0.790 | -1.641 ~ 1.249 |
| Unemployed | -1.687 | -0.188 | 0.772 | -2.185 | 0.030* | -3.200 ~ -0.173 |
| < 20,000 RMB | - | - | - | - | - | - |
| 20,000-50,000 RMB | 0.245 | 0.030 | 0.733 | 0.334 | 0.739 | -1.192 ~ 1.682 |
| 50,001-100,000 RMB | 0.228 | 0.027 | 0.937 | 0.243 | 0.808 | -1.609 ~ 2.064 |
| 100,001-200,000 RMB | -0.062 | -0.005 | 1.098 | -0.056 | 0.955 | -2.214 ~ 2.090 |
| >200,000 RMB | 0.607 | 0.019 | 2.178 | 0.278 | 0.781 | -3.663 ~ 4.876 |
| Mastectomy | - | - | - | - | - | - |
| Breast-conserving therapy | -0.889 | -0.073 | 0.754 | -1.179 | 0.240 | -2.367 ~ 0.589 |
| Unknown | 1.037 | 0.078 | 0.809 | 1.282 | 0.202 | -0.549 ~ 2.622 |
| Others | -0.089 | -0.003 | 1.779 | -0.050 | 0.960 | -3.576 ~ 3.398 |
| I | - | - | - | - | - | - |
| II | -0.729 | -0.091 | 0.591 | -1.234 | 0.219 | -1.887 ~ 0.429 |
| III | -2.201 | -0.216 | 0.767 | -2.870 | 0.005** | -3.704 ~ -0.698 |
| Unknown | -0.216 | -0.016 | 0.964 | -0.224 | 0.823 | -2.105 ~ 1.672 |
| ECOG 0 | - | - | - | - | - | - |
| ECOG 1 | -2.767 | -0.306 | 0.581 | -4.762 | 0.000** | -3.906 ~ -1.628 |
| ECOG 2 | -6.226 | -0.289 | 1.381 | -4.508 | 0.000** | -8.933 ~ -3.519 |
| ECOG 3 | -7.745 | -0.237 | 2.026 | -3.822 | 0.000** | -11.716 ~ -3.773 |
| **Potential influencing factors for Emotional well-being** | **B** | **Beta** | **Std.Err** | ***t*** | **P** | **95% CI** |
| Constants | 21.022 | - | 1.439 | 14.606 | 0.000 | 18.201 ~ 23.843 |
| Government/official/enterprise/ business | - | - | - | - | - | - |
| Self-employed/Farmer/Laborer | -1.771 | -0.146 | 1.026 | -1.726 | 0.086 | -3.782 ~ 0.240 |
| Unemployed | -3.585 | -0.306 | 1.075 | -3.336 | 0.001** | -5.691 ~ -1.479 |
| < 20,000 RMB | - | - | - | - | - | - |
| 20,000-50,000 RMB | -0.877 | -0.082 | 1.021 | -0.859 | 0.391 | -2.877 ~ 1.124 |
| 50,001-100,000 RMB | -1.627 | -0.146 | 1.304 | -1.247 | 0.214 | -4.183 ~ 0.930 |
| 100,001-200,000 RMB | -5.667 | -0.369 | 1.528 | -3.709 | 0.000** | -8.662 ~ -2.672 |
| >200,000 RMB | 0.112 | 0.003 | 3.032 | 0.037 | 0.971 | -5.831 ~ 6.054 |
| Mastectomy | - | - | - | - | - | - |
| Breast-conserving therapy | -0.740 | -0.047 | 1.050 | -0.705 | 0.482 | -2.798 ~ 1.318 |
| Unknown | 1.552 | 0.090 | 1.126 | 1.378 | 0.170 | -0.655 ~ 3.759 |
| Others | 0.421 | 0.011 | 2.476 | 0.170 | 0.865 | -4.432 ~ 5.275 |
| I | - | - | - | - | - | - |
| II | -0.568 | -0.055 | 0.822 | -0.691 | 0.490 | -2.180 ~ 1.043 |
| III | -3.757 | -0.282 | 1.067 | -3.521 | 0.001** | -5.849 ~ -1.665 |
| Unknown | 0.598 | 0.033 | 1.341 | 0.446 | 0.656 | -2.031 ~ 3.227 |
| ECOG 0 | - | - | - | - | - | - |
| ECOG 1 | -1.595 | -0.135 | 0.809 | -1.972 | 0.050 | -3.180 ~ -0.009 |
| ECOG 2 | -3.127 | -0.111 | 1.922 | -1.627 | 0.105 | -6.894 ~ 0.640 |
| ECOG 3 | -5.853 | -0.138 | 2.820 | -2.076 | 0.039* | -11.381 ~ -0.326 |
| **Potential influencing factors for Functional well-being** | **B** | **Beta** | **Std.Err** | ***t*** | **P** | **95% CI** |
| Constants | 23.522 | - | 1.281 | 18.360 | 0.000 | 21.011 ~ 26.033 |
| Government/official/enterprise/ business | - | - | - | - | - | - |
| Self-employed/Farmer/Laborer | -0.665 | -0.055 | 0.913 | -0.729 | 0.467 | -2.455 ~ 1.124 |
| Unemployed | -1.699 | -0.144 | 0.956 | -1.777 | 0.077 | -3.574 ~ 0.175 |
| < 20,000 RMB | - | - | - | - | - | - |
| 20,000-50,000 RMB | -0.733 | -0.068 | 0.909 | -0.807 | 0.421 | -2.514 ~ 1.048 |
| 50,001-100,000 RMB | -0.103 | -0.009 | 1.161 | -0.089 | 0.929 | -2.378 ~ 2.173 |
| 100,001-200,000 RMB | -1.392 | -0.090 | 1.360 | -1.023 | 0.307 | -4.058 ~ 1.274 |
| >200,000 RMB | 2.035 | 0.048 | 2.699 | 0.754 | 0.452 | -3.254 ~ 7.325 |
| Mastectomy | - | - | - | - | - | - |
| Breast-conserving therapy | 0.663 | 0.041 | 0.934 | 0.709 | 0.479 | -1.169 ~ 2.494 |
| Unknown | 2.014 | 0.116 | 1.002 | 2.010 | 0.046* | 0.050 ~ 3.979 |
| Others | -1.560 | -0.042 | 2.204 | -0.708 | 0.480 | -5.880 ~ 2.760 |
| I | - | - | - | - | - | - |
| II | -0.763 | -0.073 | 0.732 | -1.042 | 0.299 | -2.197 ~ 0.672 |
| III | -2.965 | -0.222 | 0.950 | -3.121 | 0.002** | -4.827 ~ -1.103 |
| Unknown | 2.329 | 0.128 | 1.194 | 1.951 | 0.053 | -0.011 ~ 4.669 |
| ECOG 0 | - | - | - | - | - | - |
| ECOG 1 | -4.539 | -0.383 | 0.720 | -6.304 | 0.000** | -5.950 ~ -3.128 |
| ECOG 2 | -6.035 | -0.214 | 1.711 | -3.527 | 0.001** | -9.388 ~ -2.681 |
| ECOG 3 | -12.291 | -0.288 | 2.510 | -4.896 | 0.000** | -17.211 ~ -7.371 |
| **Potential influencing factors for Endocrine symptoms** | **B** | **Beta** | **Std.Err** | ***t*** | **P** | **95% CI** |
| Constants | 64.066 | - | 1.823 | 35.152 | 0.000 | 60.494 ~ 67.638 |
| Government/official/enterprise/ business | - | - | - | - | - | - |
| Self-employed/Farmer/Laborer | 2.807 | 0.187 | 1.299 | 2.161 | 0.032* | 0.261 ~ 5.354 |
| Unemployed | 1.777 | 0.123 | 1.361 | 1.306 | 0.193 | -0.890 ~ 4.444 |
| < 20,000 RMB | - | - | - | - | - | - |
| 20,000-50,000 RMB | 0.482 | 0.036 | 1.293 | 0.373 | 0.709 | -2.051 ~ 3.016 |
| 50,001-100,000 RMB | 1.890 | 0.137 | 1.652 | 1.144 | 0.254 | -1.347 ~ 5.127 |
| 100,001-200,000 RMB | 2.032 | 0.107 | 1.935 | 1.050 | 0.295 | -1.760 ~ 5.824 |
| >200,000 RMB | 8.222 | 0.156 | 3.839 | 2.141 | 0.034* | 0.697 ~ 15.746 |
| Mastectomy | - | - | - | - | - | - |
| Breast-conserving therapy | 0.308 | 0.016 | 1.329 | 0.232 | 0.817 | -2.298 ~ 2.913 |
| Unknown | 3.397 | 0.159 | 1.426 | 2.383 | 0.018* | 0.603 ~ 6.192 |
| Others | -3.811 | -0.083 | 3.136 | -1.215 | 0.226 | -9.958 ~ 2.335 |
| I | - | - | - | - | - | - |
| II | 0.097 | 0.008 | 1.041 | 0.093 | 0.926 | -1.944 ~ 2.138 |
| III | -0.681 | -0.041 | 1.351 | -0.504 | 0.615 | -3.330 ~ 1.967 |
| Unknown | 3.138 | 0.140 | 1.698 | 1.848 | 0.066 | -0.191 ~ 6.467 |
| ECOG 0 | - | - | - | - | - | - |
| ECOG 1 | -4.794 | -0.329 | 1.024 | -4.681 | 0.000** | -6.802 ~ -2.787 |
| ECOG 2 | -2.430 | -0.070 | 2.434 | -0.998 | 0.319 | -7.200 ~ 2.341 |
| ECOG 3 | 1.496 | 0.028 | 3.571 | 0.419 | 0.676 | -5.503 ~ 8.495 |
| **Potential influencing factors for FACT-ES** | **B** | **Beta** | **Std.Err** | ***t*** | **P** | **95% CI** |
| Constants | 158.202 | - | 4.778 | 33.113 | 0.000 | 148.838 ~ 167.566 |
| Government/official/enterprise/ business | - | - | - | - | - | - |
| Self-employed/Farmer/Laborer | -0.657 | -0.015 | 3.406 | -0.193 | 0.847 | -7.332 ~ 6.018 |
| Unemployed | -7.138 | -0.163 | 3.567 | -2.001 | 0.047* | -14.129 ~ -0.147 |
| < 20,000 RMB | - | - | - | - | - | - |
| 20,000-50,000 RMB | -1.525 | -0.038 | 3.388 | -0.450 | 0.653 | -8.165 ~ 5.116 |
| 50,001-100,000 RMB | -1.084 | -0.026 | 4.329 | -0.250 | 0.803 | -9.570 ~ 7.402 |
| 100,001-200,000 RMB | -7.142 | -0.125 | 5.072 | -1.408 | 0.161 | -17.083 ~ 2.799 |
| >200,000 RMB | 12.634 | 0.080 | 10.064 | 1.255 | 0.211 | -7.091 ~ 32.360 |
| Mastectomy | - | - | - | - | - | - |
| Breast-conserving therapy | 0.145 | 0.002 | 3.485 | 0.042 | 0.967 | -6.685 ~ 6.975 |
| Unknown | 9.183 | 0.143 | 3.737 | 2.457 | 0.015* | 1.857 ~ 16.508 |
| Others | -6.724 | -0.049 | 8.220 | -0.818 | 0.414 | -22.836 ~ 9.387 |
| I | - | - | - | - | - | - |
| II | -2.575 | -0.066 | 2.730 | -0.943 | 0.347 | -7.926 ~ 2.775 |
| III | -11.767 | -0.237 | 3.542 | -3.322 | 0.001** | -18.710 ~ -4.824 |
| Unknown | 6.490 | 0.096 | 4.452 | 1.458 | 0.147 | -2.236 ~ 15.216 |
| ECOG 0 | - | - | - | - | - | - |
| ECOG 1 | -16.573 | -0.377 | 2.685 | -6.173 | 0.000** | -21.835 ~ -11.311 |
| ECOG 2 | -25.596 | -0.244 | 6.381 | -4.012 | 0.000** | -38.102 ~ -13.091 |
| ECOG 3 | -33.554 | -0.212 | 9.361 | -3.584 | 0.000** | -51.902 ~ -15.206 |

EWB=emotional wellbeing, FWB=functional wellbeing, PWB=physical wellbeing, SWB= social/family wellbeing, ES=Endocrine symptoms, NS=not significant; R^2^=coefficient of determination, β=regression coefficients, ECOG=Eastern Cooperative Oncology Group

*P<0.05, **P<0.01
